# Meniscus sizing using three-dimensional models of the ipsilateral tibia plateau based on CT scans – an experimental study of a new sizing approach

**DOI:** 10.1186/s40634-020-00252-8

**Published:** 2020-05-27

**Authors:** Silvan Beeler, Lazaros Vlachopoulos, Lukas Jud, Reto Sutter, Tobias Götschi, Philipp Fürnstahl, Sandro F. Fucentese

**Affiliations:** Department of Orthopaedic Surgery, University of Zurich, Balgrist University Hospital, Forchstrasse 340, 8008 Zurich, Switzerland

**Keywords:** Knee, Meniscus sizing, Meniscus medial, Meniscus lateral, Three-dimensionally segmentation, Magnetic resonance, MRI scans, Inter-observer variation

## Abstract

**Purpose:**

Selection of a meniscus allograft with a similar three-dimensional (3D) size is essential for good clinical results in meniscus allograft surgery. Direct meniscus sizing by MRI scan is not possible in total meniscectomy and indirect sizing by conventional radiography is often inaccurate. The purpose of this study was to develop a new indirect sizing method, based on the 3D shape of the ipsilateral tibia plateau, which is independent of the meniscus condition.

**Methods:**

MRI and CT scans of fifty healthy knee joints were used to create 3D surface models of both menisci (MRI) and tibia plateau (CT). 3D bone models of the proximal 10 mm of the entire and half tibia plateau (with / without intercondylar area) were created in a standardized fashion. For each meniscus, the best fitting “allograft” couple out of all other 49 menisci were assessed by the surface distance of the 3D meniscus (best available allograft), of the 3D tibia plateau (3D-CT) and by the radiographic method of Pollard (2D-RX).

**Results:**

3D-CT sizing was significantly better by using only the half tibia plateau without the intercondylar area (*p* < 0.001). But neither sizing by 3D-CT, nor by 2D-RX could select the best available allograft. Compared to 2D-RX, 3D-CT sizing was significantly better for the medial, but not for the lateral meniscus.

**Conclusions:**

Automatized, indirect meniscus sizing using the 3D bone models of the tibia plateau is feasible and more precise than the previously described 2D-RX method.. However, further technical improvement is needed to select always the best available allograft.

## Background

Partial or total meniscectomy is often a valuable treatment option to improve the symptoms of patients with meniscal tears. However, the biomechanical properties of the meniscus are thereby lost and early osteoarthritis or residual pain might occur [16].

Meniscus allograft transplantation has become a powerful tool in the treatment of postmeniscectomy syndrome in young patients with proven clinical and functional efficacy [21].

Overall, best biomechanical and clinical results could be achieved by the selection of an allograft with a similar three-dimensional (3D) meniscus shape [4, 10, 12, 19, 20, 25]. Nevertheless so far, the 3D shape of the meniscus was completely ignored and meniscus sizing performed only by meniscal width and length in conventional radiography [17, 26], MRI [7, 11, 15, 22] or – rarely – by CT [5, 9, 14]. Although good average sizing values are reported in literature, inaccuracy of sizing is still a relevant problem and has to be improved. The ignored 3D shape of the meniscus might lead to an inaccurate meniscus size selection, regrettably corresponding with clinical experience and confirmed by recently published data [1, 6, 9, 13, 14, 17, 22, 23].

So far, MRI sizing by with and length of the contralateral healthy meniscus is considered to be the gold standard [13, 18]. But recently, a 3D meniscus sizing method was proposed by using the contralateral meniscus as a template, based on the closest mean surface distance [1, 2]. Herewith, the superiority of 3D sizing compared to sizing only by meniscal width and length has already be shown. However, this sizing method is strongly dependent on a healthy contralateral meniscus and time-consuming manual MRI segmentation of the menisci.

The aim of this study was, therefore, to develop a new indirect meniscus sizing method, that should be independent of the meniscus condition, fully automatized with validated technologies and should improve the accuracy of the entire 3D meniscus shape selection. We expected a more accurate sizing using the entire 3D information of the tibia plateau, compared to sizing only by meniscal width and length.

## Methods

The methodological part is composed by three parts: Part 1 with focus on the basic material, imaging and measurement methods. Part 2 with description and implementation of the 3D CT sizing method. And part 3 with analysis and validation of the sizing methods. Ethical approval was granted by Kantonale Ethikkommission of Zurich, Switzerland (BASEC-Nr. 2018–00856), and informed consent was obtained from all individual participants included in the study. A written consent for publication of their clinical details and/or clinical images was obtained from each patient. The datasets used and analyzed during the current study are available from the corresponding author on reasonable request. There are no conflicts of interest.

### Part 1: Material/imaging/measurements

#### Material

We retrospectively analyzed fifty unilateral knee joints of fifty patients with patellofemoral disorders. Inclusion criteria were mature skeletal age (completely closed growth plate of femoral and tibial knee epiphyses in MRI), healthy meniscus (no tears, degeneration or extrusion), no tibio-femoral osteoarthritis (Kellgren & Lawrence grade 0), available magnetic resonance imaging (MRI; sagittal/coronal/axial plane; slice thickness of 3 mm), computer tomography (CT; axial; slice thickness of ≤2 mm) and conventional radiography (RX; antero-posterior (AP) / medio-lateral (LAT)).

Mean age of all fifty patients was 27.6 years (16–46 years). There were 18 left and 32 right knee joints of 35 female and 15 male patients.

#### Imaging

All radiographs (RX) were performed in a standard fashion, as already described in a previous paper [1]. Plain anteroposterior (AP) and lateral (LAT) view were acquired with a detector-to-tube distance of 1.15 m and using calibrators for the correction of magnification (Ysio, Siemens Healthcare, Erlangen, Germany).

All CT scans were performed on a 64-slice CT scanner (Philips Brilliance 64, Philips Healthcare, or Somatom Definition AS, Siemens Healthcare) using our standard protocol for knee joints. Technical specifications: tube voltage 120 kV, tube current 250 mAs, collimation 64 × 0.625 mm, and rotation time 0.5 s. Axial images were reconstructed with 1 mm slice thickness.

All MRI scans consisted of sagittal, coronal and axial sequences and were performed on a 3.0 T magnet (Skyra-fit, Siemens Healthcare, Erlangen, Germany) with a dedicated knee coil in supine position with stretched knee, as a part of the standard MRI procotol [2].

#### Measurements

##### 3D surface models (meniscus, tibia plateau):

**Fig. 1:**
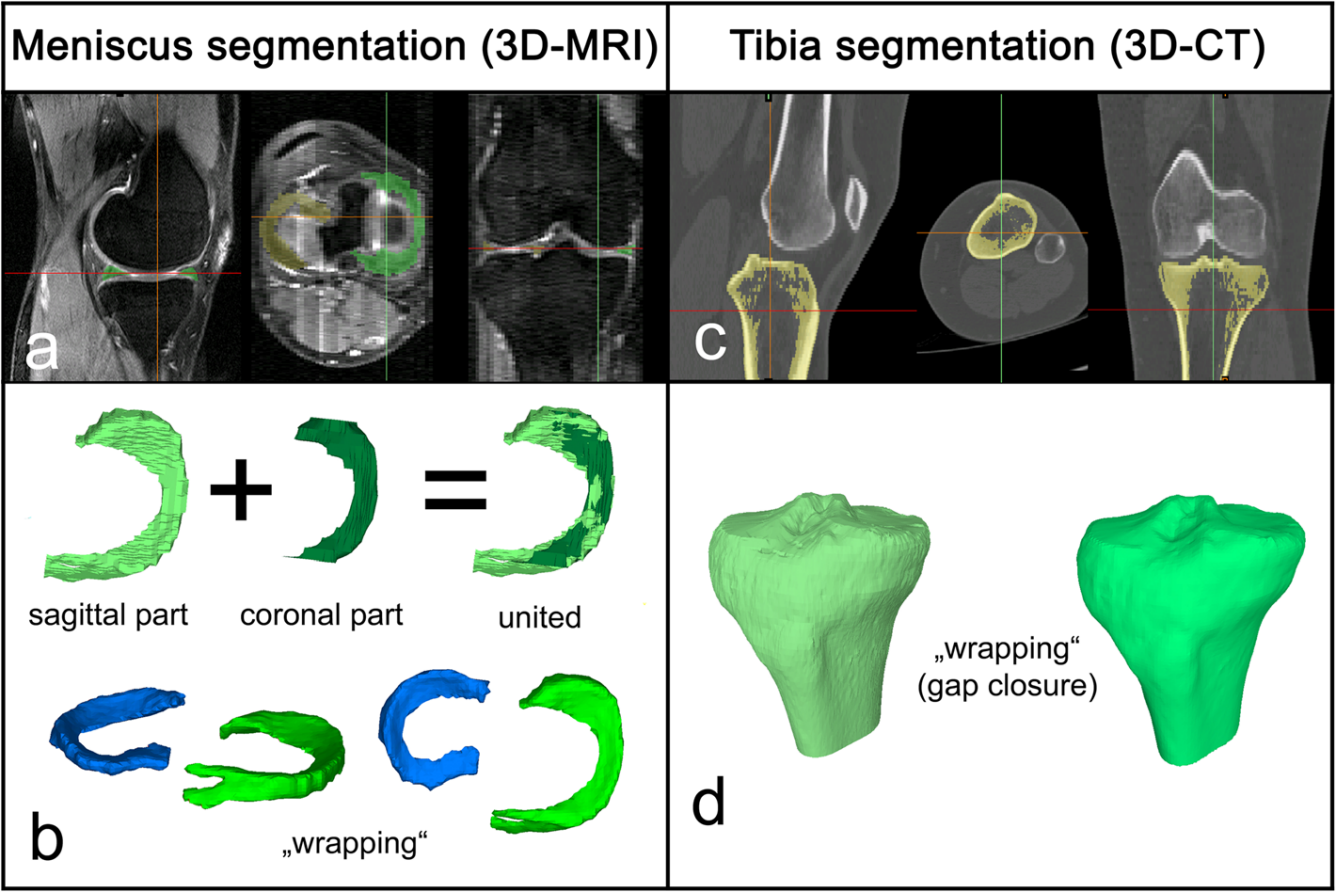
3D surface models: **a**. *Step 1 (Meniscus): Segmentation was performed in sagittal and coronal slides by manually annotation of all meniscus tissue.***b**. *Step 2 (Meniscus): Three-dimensional sagittal and coronal meniscus parts were merged. If there was a shift due to patients movement during acquisition of the different reconstructions, manual realignement has to be performed first. Wrapping of the meniscus surface models was done by using the functionallity of the 3-matic software.****c****. Step 1 (Tibia): Segmentation was performed in axial, coronal and sagittal reconstructions by semi-automatic annotation of all tibia bone.***d**. *Step 2 (Tibia): Wrapping of the tibia surface models was done by using the functionallity of the 3-matic software*

1. Meniscus: *(3D-MRI)*
  Segmentation: Meniscus segmentation was done manually by two trained orthopaedic surgeons (S.B., L.J.) in sagittal and coronal slides (bi-planar) with the Materialise Interactive Medical Control System (MIMICS) 3D reconstruction software program 18.0 (Materialise, Leuven, Belgium), as described in a previous paper [2]. The 3D surface models of the final meniscal models were smoothed using the wrapping functionallity of the software with gap closing distance 0.0 and smallest detail 1.0 (Fig. 1a, b).
  Mirroring: All 3D surface models of the left meniscus were mirrored to a right meniscus.
2. Tibia plateau: *(3D-CT)*
  Segmentation: Tibia plateau segmentation was done semi-automated in axial, coronal and sagittal reconstructions with MIMICS. The segmentation was controlled and – if necessary due to the 2 mm slide thickness – slightly corrected by two trained orthopaedic surgeons (S.B., L.J.). The 3D surface models of the cortical layer were created and the same wrapping functionallity was used as before (Fig. 1c, d).
  Mirroring: All 3D surface models of the left tibia pleateau were mirrored to a right tibia plateau.

##### Meniscus dimensions (width, length, height):

**Fig. 2:**
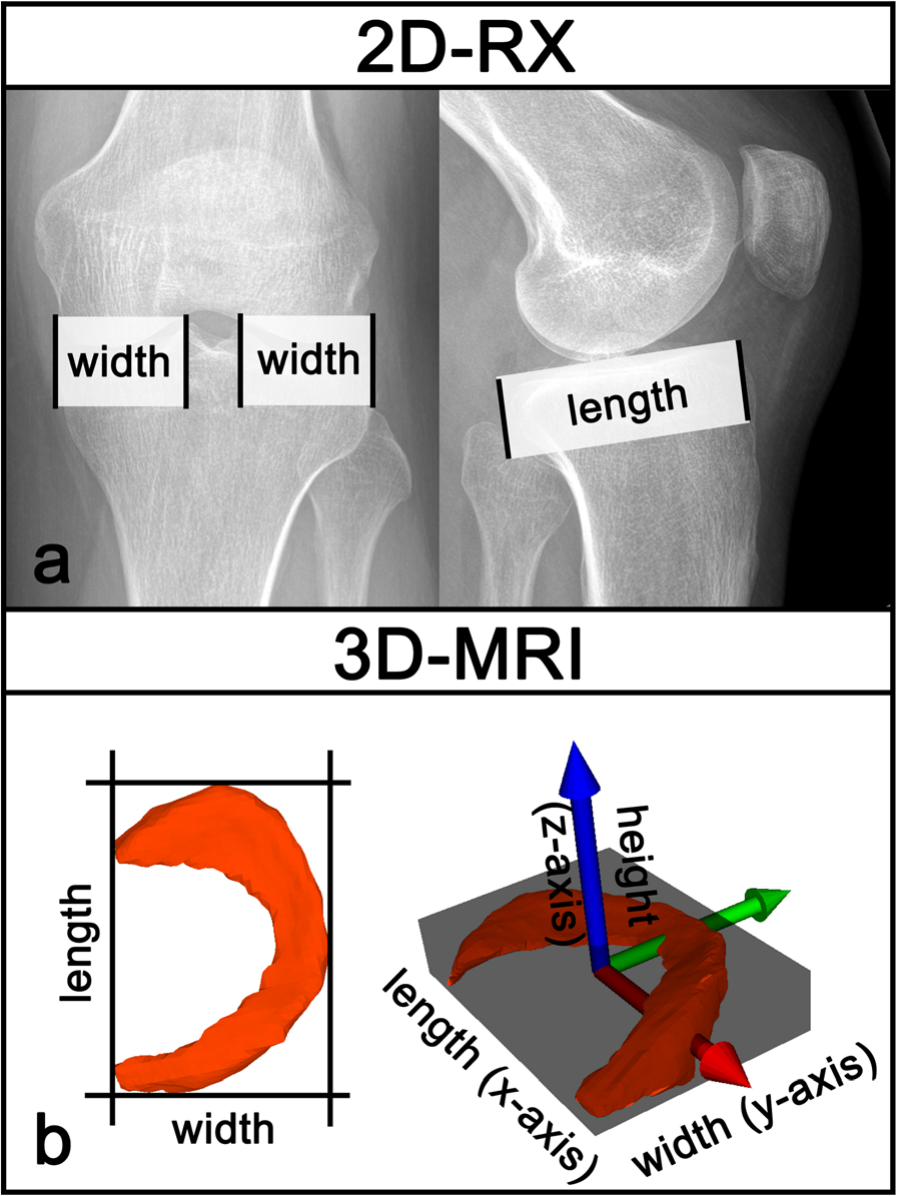
Measurement meniscus dimensions in 2D-RX, 2D-MRI and 3D-MRI. **a***2D-RX: Meniscus length = Distance between the anterior surface of the tibia above the tuberosity to the posterior margin of the tibial plateau, perpendicular to the joint line. Multiplication by 0.8 for medial and 0.7 for lateral meniscus. Meniscus width = Distance from the margin of the tibial metaphysis to the medial, respectively lateral tibial eminences, perpendicular to the joint line.***b***3D-MRI: A best fitting three-dimensional box was placed around the meniscus. X-axis was parallel to a line between anterior and posterior meniscus root. X-axis = meniscus length, Y-axis = meniscus width, Z-axis = meniscus height*

1. Conventional radiography: (2D-RX) (Fig. 2a)
  Meniscal width and length was measured based on the method proposed by Pollard [17]. Meniscus width was measured in the AP view as the distance from the margin of the tibial metaphysis to the medial, respectively lateral tibial eminences, perpendicular to the joint line. Meniscus length was measured in the lateral view as the distance between the anterior surface of the tibia above the tuberosity to the posterior margin of the tibial plateau, perpendicular to the joint line. All distances were measured twice by two different readers (S.B., L.J.) and the average value of both were used for further calculations.
2. Three-dimensional MRI: (3D-MRI) (Fig. 2b)
  Width, length and height was measured as described in a previous paper [2]. An oriented bounding box (OBB) was calculated from all model points. The OBB is the minimal-volume rectangular box fully enclosing the meniscal model. The box was now rotated around the z-axis (Fig. 2b, blue axis) until the box was oriented parallel to the anterior and posterior meniscus root (Fig. 2b, red axis). The box was now adjusted in width, length and height until the meniscus was fully enclosing by the box. Length (x-axis), width (y-axis) and height (z-axis) of the meniscus can now be defined by the lengths of the box.

### Part 2: 3D-CT sizing

**Fig. 3:**
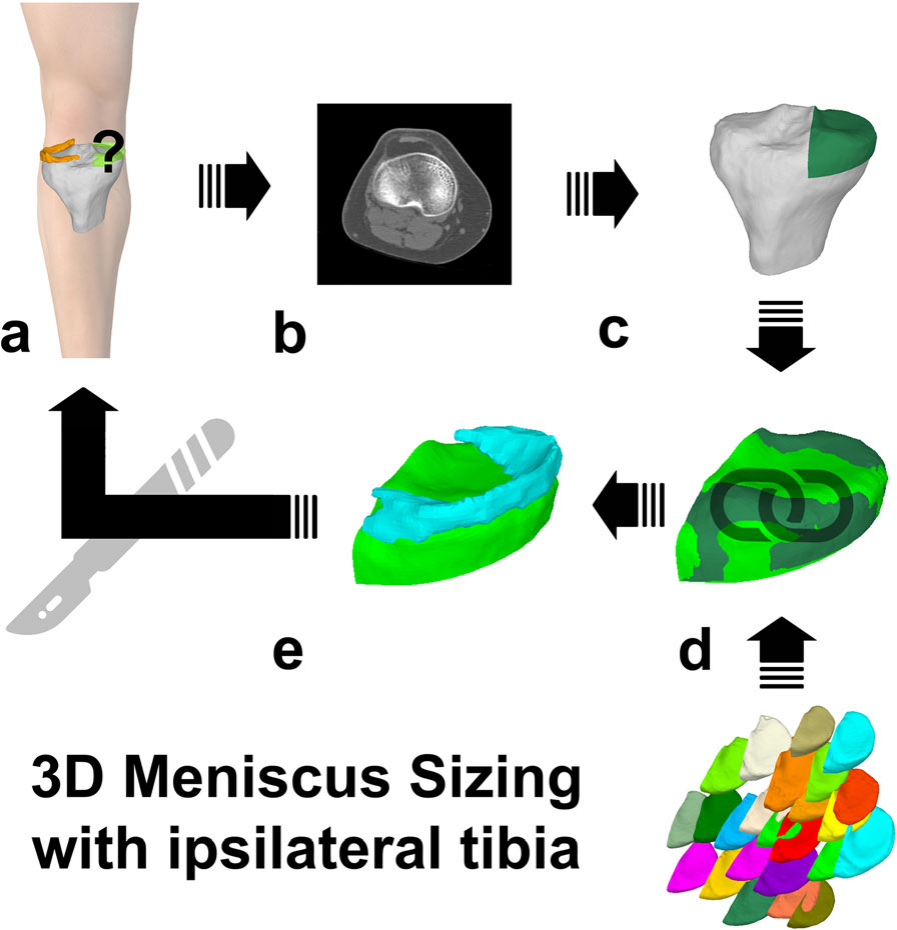
3D meniscus sizing with ipsilateral tibia plateau. **a***Right knee with missing medial meniscus*. **b***CT scan of the ipsilateral side*. **c***Tibia plateau segmentation and disc cutting (see also* Fig. 4*)*. **d***Tibia plateau matching with all tibia plateaus of the tissue bank by mean and maximum surface distances (see also* Fig. 4*)*. e *Selection of best fitting tibia plateau with attached best fitting meniscus for meniscus allograft surgery*

3D-CT sizing is an indirect sizing method, based on 3D surface bone models of parts of the tibia plateau. The sizing method is based on the assumption that an equal tibia plateau contains an equal shaped meniscus, similar to an imprint of a trace. Thereby, not the most similar meniscus is ordered from the tissue bank, but the most similar tibia plateau – with indirectly contained most similar meniscus (Fig. 3).

#### Creation of the different predefined 3D models of the tibia plateau

**Fig. 4:**
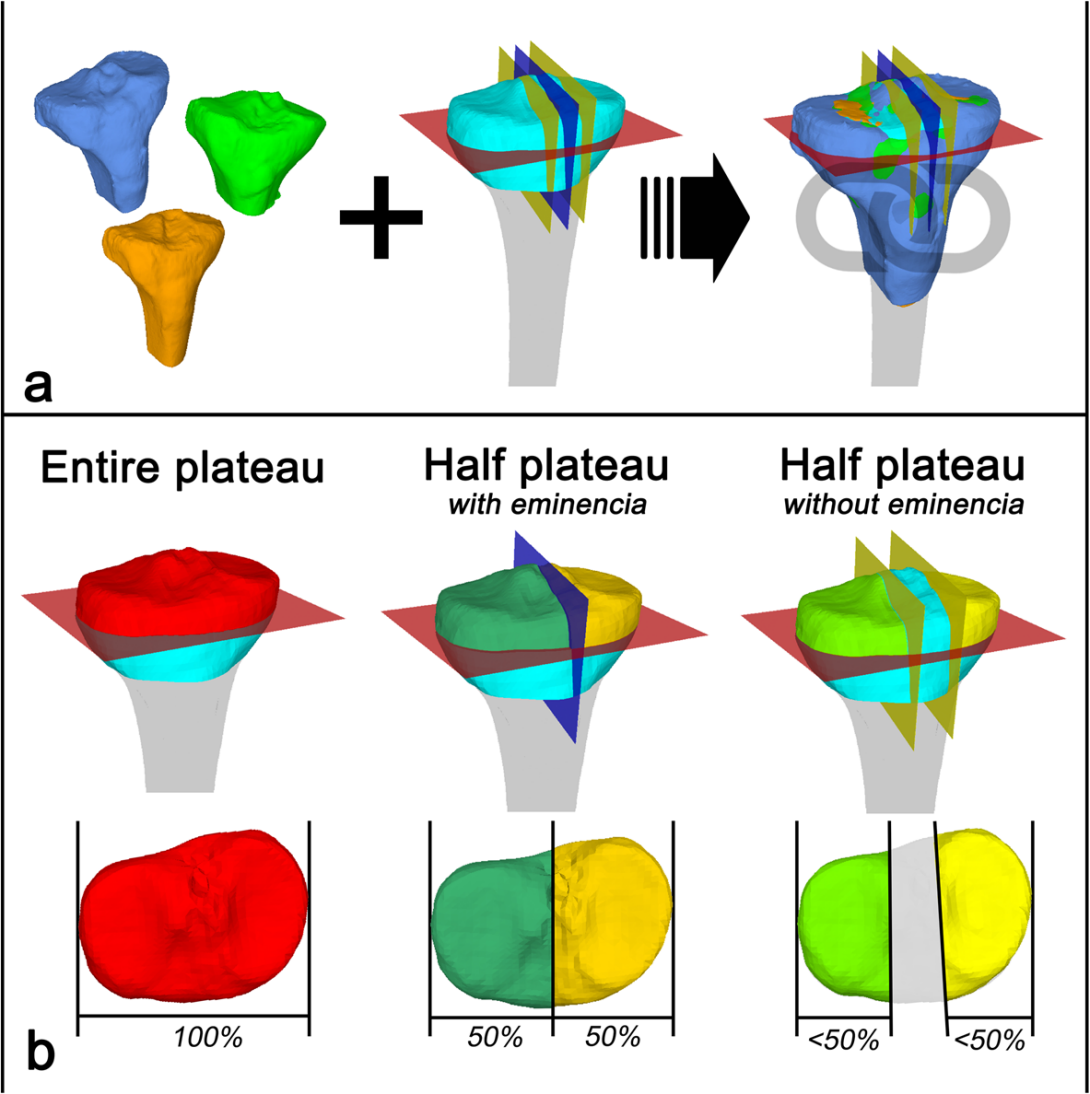
Creation of the different predefined 3D models of the tibia plateau. **a***Different proximal tibia plateaus (left) are automatically superimposed (right) on the average right tibia template (middle).***b***Entire plateau = Cutting with the axial cutting plane. Half plateau with eminence = Cutting with the axial and centered sagittal cutting plane. Half plateau without eminence = Cutting with the axial and medial/lateral cutting plane with exclusion of the intertrochanteric region*

All 3D bone models of the tibia plateaus were imported into the in-house developed planning software CASPA (Computer Assisted Surgery Planning Application, Balgrist CARD AG). An average right tibia template of 3.5 cm length with preconfigured cutting planes served as a target alignment model for automatized adjustment and standardized creation of the different tibia plateaus. Following cutting planes were first defined with regard to the tibia plateau template: A first axial cutting plane (Fig. 4; red cutting plane) was set 1 cm distal and parallel to the medial and lateral tibia plateau. A second sagittal cutting plane (Fig. 4; blue and yellow cutting plane) was set perpendicular to the first and parallel to the orientation of medial and lateral intercondylar tubercles and tibia plateau.

For tibia plateau sizing, we selected three different configurations:

1. Entire tibia plateau (100%): The proximal tibia was only cut with the first, axial cutting plane (Fig. 4; red cutting plane).
2. Half tibia plateau with intercondylar area (50%): The proximal tibia plateau was cut with the first and second cutting plane. Thereby, the second cutting plane was set centered to the medial and lateral plateau dimensions (Fig. 4; blue cutting plane).
3. Half tibia plateau without intercondylar area (< 50%): The proximal tibia plateau was cut with the first and second cutting plane. Now, the second two cutting planes were set once through the medial and once through the lateral intercondylar eminence (Fig. 4; medial and lateral yellow cutting planes).

#### 3D-CT sizing by closest surface points distance

The similarity of two 3D models can be quantified by the closest surface points distance [2]. After automatically superimposing using the iterative closest point (ICP) algorithm, mean (MeSD) and maximum distances of all surface points (MaSD) of one model to the closest points of the second model – and vice versa – can be calculated. For further calculations, only the highest values are used according to the Hausdorff distance [2]. This process can now be repeated between all 3D models. The best fitting pair can be found by the lowest mean and maximum surface distances.

### Part 3: validation

Unlike previously sizing methods, that allowed directly comparison of measured width and length of the original and the calculated meniscus dimensions, the 3D-CT sizing method allows only indirect measurements. Therefore, we solved this problem by a simulation, as already used in a previous study about 3D-MRI meniscus allograft sizing [1].

In a first step, for each meniscus, the best fitting “allograft” was selected by all three methods (3D-MRI, 3D-CT, 2D-RX). Thereby, the other 49 menisci served as an imaginary “tissue bank”. All measurements were made separately for the medial and lateral meniscus.

1. 3D-MRI meniscus sizing by surface distance (best possible allograft) (3D-MRI):

Direct meniscus sizing by the lowest mean surface distance was assessed to select the best possible 3D fitting meniscus in our “tissue bank” of 49 different menisci. Thereby, meniscus “1” was matched with meniscus “2”, “3”, …. to “50”. The best fitting couple (1 × 49 possibilities) was selected and used for further calculations. Then, meniscus “2” was matched with meniscus “1”, “3”, …. to “50”, and again, the best fitting couple was selected. This was repeated for all fifty menisci. Together, there were 2′450 (50 × 49) possibilities. All calculations could be done full automatized by an in house-developed application.

Mean/maximum surface distance and the differences of width/length/height between the best fifty couples were used for comparison of the different sizing methods.

2.) 3D-CT tibia sizing by surface distance (3D-CT):

Indirect meniscus sizing by the tibia plateau was done in two steps. First, the corresponding tibia plateau (tibia) of the original meniscus served as template. Tibia “1” (of corresponding meniscus “1”) was matched with tibia “2”, “3”, …. to “50”. The best fitting couple (49 possibilities) was selected. Then, tibia “2” was matched with tibia “1”, “3”, …. to “50” and the best couple was selected. This was repeated for all fifty tibia plateaus (2′450 possibilities). Second, the selected best fitting tibias were replaced by the corresponding menisci.

Mean/maximum surface distance and the differences of width/length/height between the best fifty couples were used for comparision of the different sizing methods.

This procedure was done with the 1) entire tibia plateau, 2) half tibia plateau with intercondylar area and 3) half tibia plateau without intercondylar area.

3.) 2D-RX tibia sizing by width/length according to Pollard [17] (2D-RX):

Width and length were measured as described above in conventional radiography of all fifty knee. The best fitting meniscus pair for meniscus “1” was selected out of the three-dimensional measured width/length of the other 49 menisci. Thereby, width and length were equally weighted and selected by the lowest error sum of squares: (Width_Sized_ - Width_Allograft_)^2^ + (Length_Sized_ - Length_Allograft_)^2^ (2′450 possibilities).

Mean/maximum surface distance and the differences of width/length/height between the best fifty couples were used for comparison of the different sizing methods.

Finally, the best fifty selected menisci of each method were compared according to the MeSD, MaSD and the differences of width/length/height. Further, outliers were analyzed as a mismatch between the “original” and “selected meniscus” of > 5 mm in width, > 5 mm in length, > 4 mm in height or > 5 mm MaSD [1].

### Statistics

Average performance of the different CT-based matching techniques was compared using repeated measures ANOVA with Greenhouse-Geisser correction where applicable. The CT technique with the best average performance measured by mean surface distance between the original meniscus and the chosen allograft was further compared against the other applied modalities. Here average performance (i.e. accuracy) in all included proximity measures as well as consistency (i.e. precision) of the different methods was assessed. The former was conducted analogously to the method described above and the latter was determined using Levene’s test for equality of variance. The assessed methods were then compared in pairwise manner using paired t-tests to analyze consistency and F-testing to analyze accuracy. Significance levels were Bonferroni-corrected.

## Results

### Meniscus sizes

The medial meniscus was on average 31.9 mm (range 24.8–38.2 mm, SD 3.14) wide, 46.4 mm (range 37.4–55.0 mm, SD 46.4) long and 8.9 mm (range 6.1–13.5 mm, SD 1.51) high. The lateral meniscus was on average 32 mm (range 24.8–39.8 mm, SD 3.52) wide, 35.3 mm (range 29.4–42.4 mm, SD 3.01) long and 9.7 mm (range 7.8–13.2 mm, SD 1.37) high.

### 3D CT sizing

The half tibia plateau without the intercondylar eminence yielded the most reliable sizing. Significant differences could be found in mean surface distance (F(2) = 13.075, *p* < 0.001) and maximum surface distance (F(1.780) = 8.491, *p* = 0.001) of the medial meniscus and in mean surface distance (F(1.789) = 14.375, p < 0.001) and maximum surface distance (F(2) = 7.606, p = 0.001) of the lateral meniscus. All results can be seen in Table 1.

**Table 1:** 3D-CT Sizing by entire and half tibia plateau

	**Entire plateau**		**Half plateau** **with eminence**		**Half plateau** **without eminence**	
***(mm)***	**MM**	**LM**	**MM**	**LM**	**MM**	**LM**
**Mean Surface Distance**						
Mean	1.32	1.39	1.23	1.29	0.96	1.02
Min-Max	0.71–2.57	0.73–2.84	0.74–2.48	0.74–2.32	0.67–1.44	0.71–1.46
Std	0.447	0.495	0.452	0.399	0.180	0.216
**Maximum Surface Distance**						
Mean	5.51	5.58	5.11	5.29	4.36	4.61
Min-Max	2.35–10.1	3.13–8.58	2.97–9.9	3.07–8.79	2.09–8.2	2.87–7.96
Std	1.86	1.61	1.89	1.60	1.22	1.08

*MM* Medial Meniscus, *LM* Lateral Meniscus

### Validation

Allograft selection by the 3D surface of the target meniscus (3D MRI) served as the gold standard (= best possible meniscus allograft out of the imaginary tissue bank of 49 different menisci). With this quantity of only 49 different allografts, it was possible to select a relatively similar allograft with a MeSD less than 1.12 and MaSD less than 5.33 mm. And there were no meniscus mismatch (outliers) > 5 mm in width/length and > 4 mm in height.

**Fig. 5:**
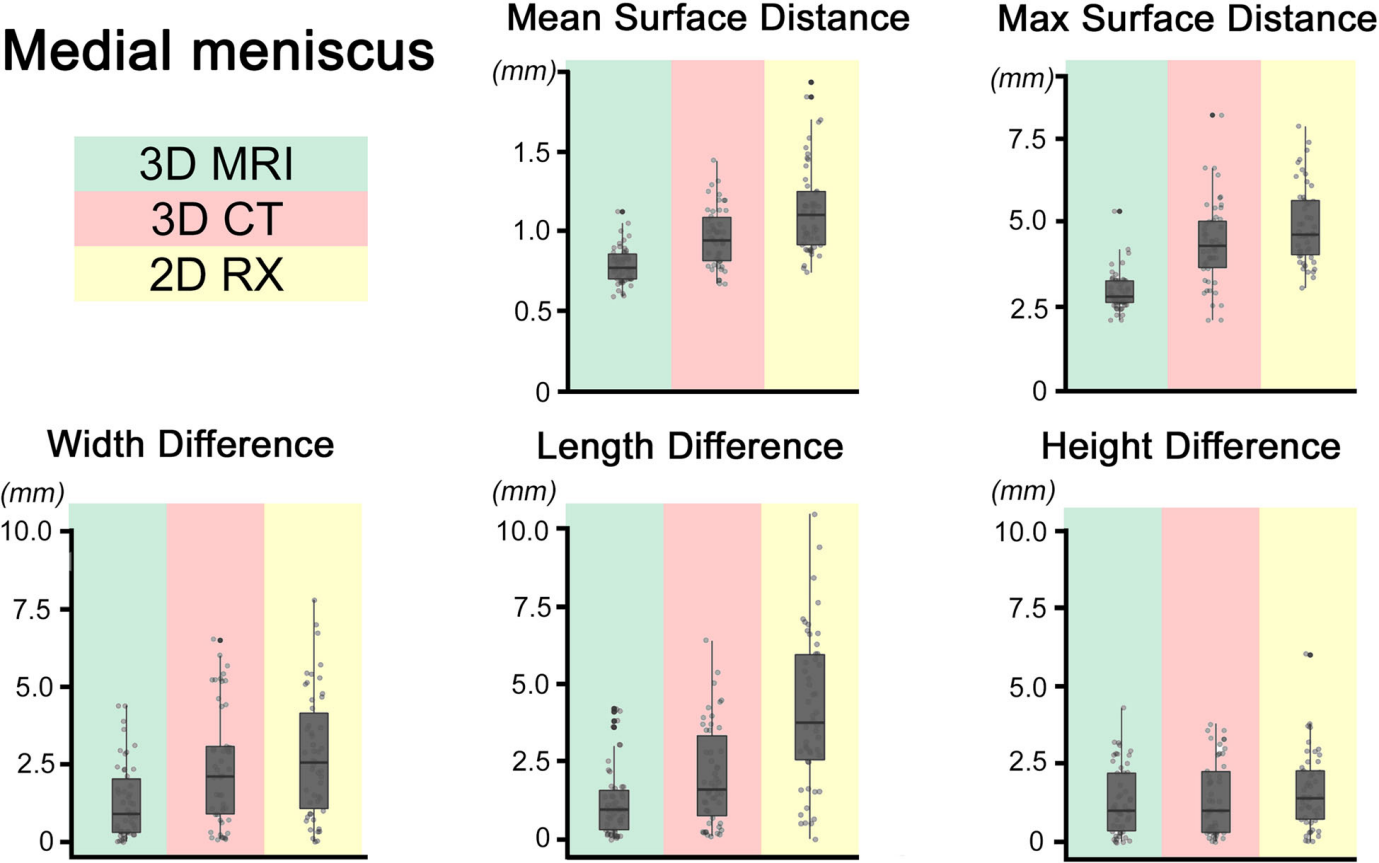
Medial Meniscus. *Boxplots for medial meniscus. The box represents the middle 50% of values. The box represents the middle 50% of values. The ends of the whiskers indicate 1.5 times the IQR (inter quartile range) between the lower and upper quartile, and outliers are denoted with a circle*

**Fig. 6:**
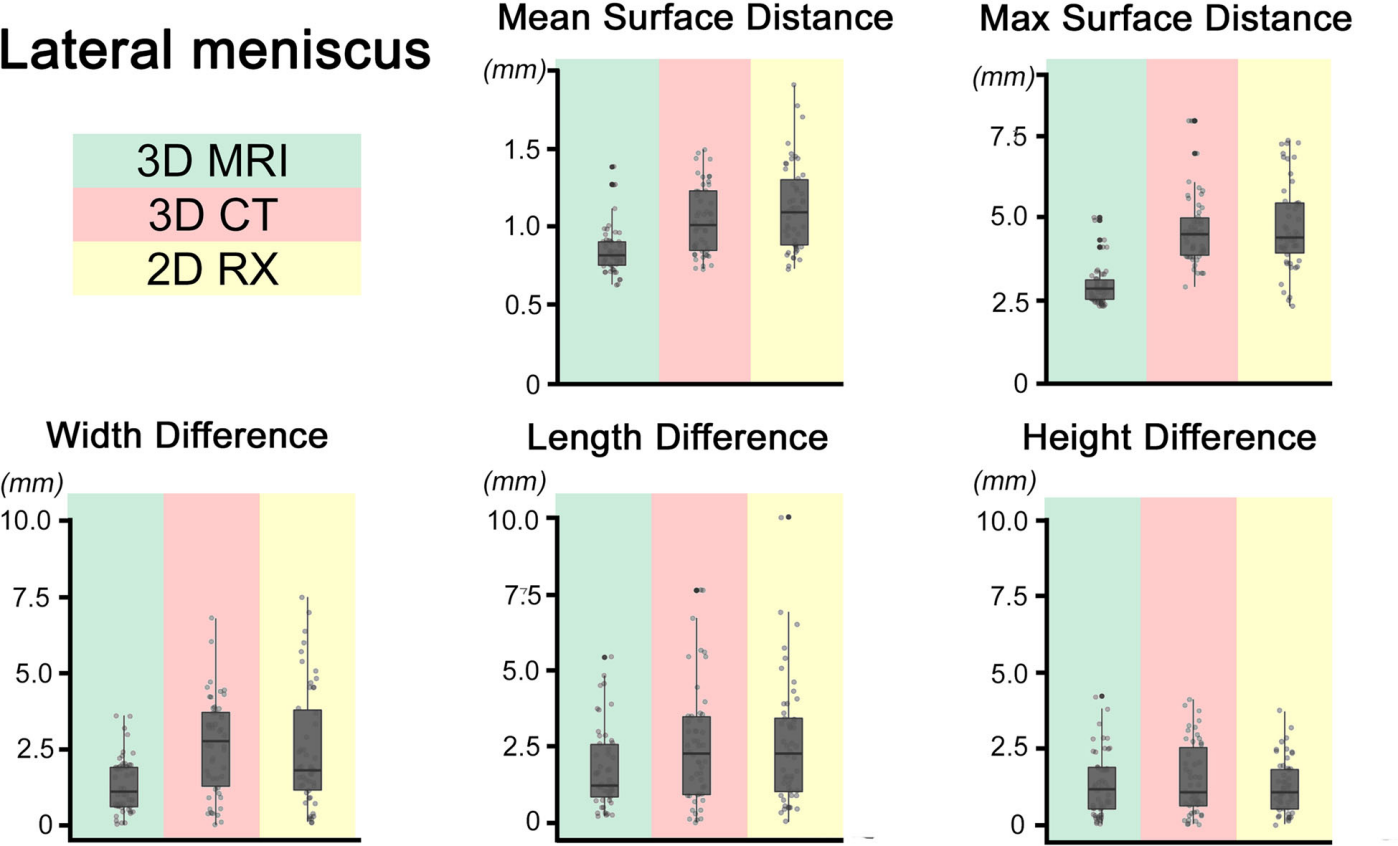
Lateral Meniscus: *Boxplots for lateral meniscus. The box represents the middle 50% of values. The ends of the whiskers indicate 1.5 times the IQR (inter quartile range) between the lower and upper quartile, and outliers are denoted with a circle*

Neither the 3D-CT nor 2D-RX sizing method could detect in each case the best fitting allograft (3D-MRI). (see Tables 2 and 3, Figs. 5 and 6).

**Table 2:**
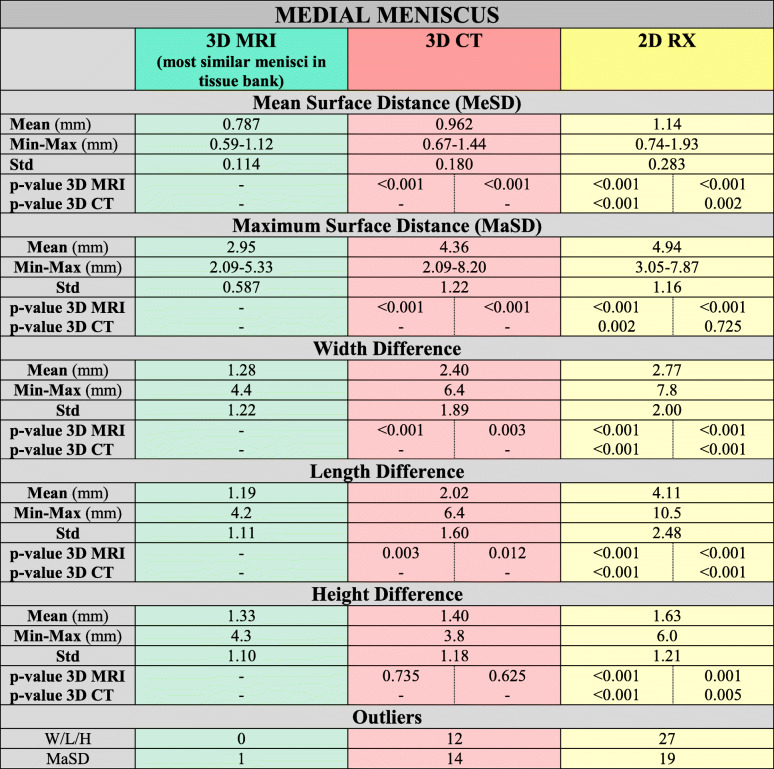
Sizing results of medial meniscus

*Mean = Mean values in mm. Min-Max = Minimum and Maximum value. SD = Standard Deviation. P-value = Displayed are the p-values of pairwise comparison of all applied methods. The left cell displays the p-value for the comparison of mean performance whereas the right cell displays p-values for differences in variance of the methods. Significance level after Bonferroni-correlation: a = 0.0083. Outliers: W = Width difference > 5 mm and L = Length difference > 5 mm and H = Height difference > 4 mm / MaSD = Maximal surface distance > 5 mm*

**Table 3:**
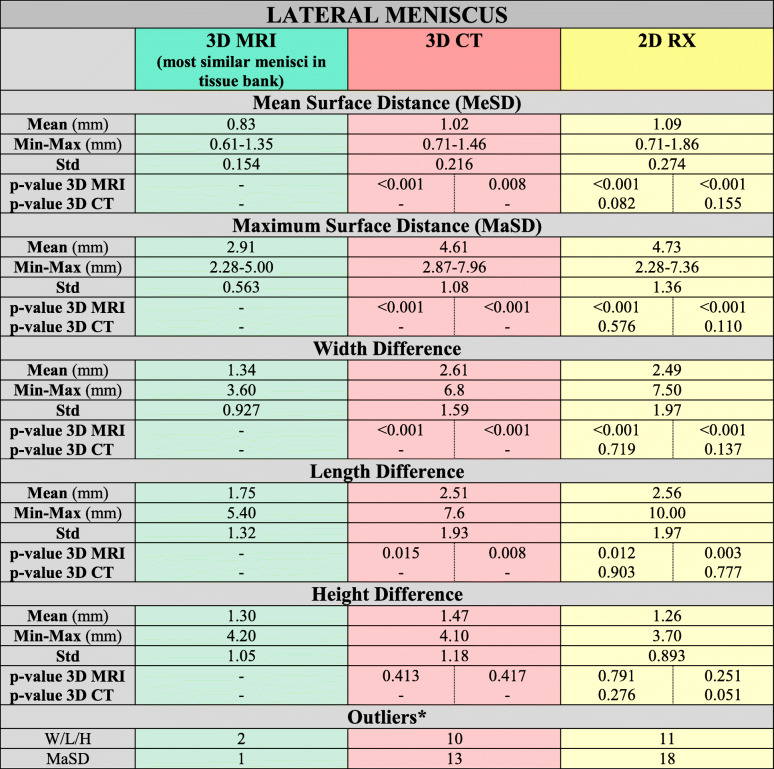
Sizing results of lateral meniscus

*Mean = Mean values in mm. Min-Max = Minimum and Maximum value. SD = Standard Deviation. P-value = Displayed are the p-values of pairwise comparison of all applied methods. The left cell displays the p-value for the comparison of mean performance whereas the right cell displays p-values for differences in variance of the methods. Significance level after Bonferroni-correlation: a = 0.0083. Outliers: W = Width difference > 5 mm and L = Length difference > 5 mm and H = Height difference > 4 mm / MaSD = Maximal surface distance > 5 mm*

Meniscus sizing by 3D-CT was significantly better than 2D-RX considering the average values (p = at least 0.002). There were no significant differences for the lateral meniscus. Outliers could be reduced by 56% for medial and 9% for lateral meniscus, compared to the 2D-RX method.

All results are listed in Tables 2 and 3 and Figs. 5 and 6.

## Discussion

The most important finding of the present study is, that automatized, indirect meniscus sizing by the 3D tibia plateau is feasible and more precise than by the radiographic method of Pollard. To our knowledge, this is the first attempt to use the 3D surface of the tibia plateau for indirect meniscus allograft sizing. It is based on the hypothesis, that the menisci of two similar tibia plateaus are similar in the 3D size. Thereby, not the allograft itself has to be ordered. Rather, the surgeon receives an equal tibia plateau with attached perfect fitting meniscus allograft. This method would be especially advantageous if meniscus fixation is performed by bone plugs [20] or combined osteochondral allograft with meniscal allograft transplantation [8]. An extension of this sizing method could be the possibility, to create a digital database of surface models of different tibia plateaus with corresponding meniscus models. 3D meniscus surface models, which could be needed as a template for biomimetic 3D printed scaffolds [24, 27] or 3D meniscus tissue engineering [3] could now be selected indirectly over the tibia plateau with a CT scan of the ipsilateral tibia.

With the currently used cutting planes, sizing with the entire plateau failed completely, while sizing without the intercondylar eminence could show the best results. As a conclusion, the medial and lateral tibia plateau do not correlate well and the better the anatomical separation of the plateau, the better would also be the sizing. While the meniscal base is defined well by the edge of the tibia plateau, the meniscus roots do not have a directly visible bony landmark. Nevertheless, the medial and lateral intercondylar tubercles with attached anterior and posterior cruciate ligaments are used as “indirect” landmarks to define the ends of the meniscus roots in bony based meniscal sizing methods [5, 9, 14, 17, 26]. Therefore, the intercondylar region between the medial and lateral intercondylar tubercles has been excluded on the average tibia plateau template.. Herewith, the accuracy could be increased – compared to the method of Pollard [17]– for medial meniscus significantly, but not for lateral meniscus. Further, outliers could be reduced by 56% for medial and 9% for lateral meniscus. Nevertheless, there is still great potential for increasing the results of this study, because neither sizing by 3D-CT, nor by 2D-RX could select in each case the best possible allograft (3D-MRI). Although the differences seem to be small (mean surface distance (MeSD) 0.18–0.35 mm, maximum surface distance (MaSD) 1.25–1.99 mm), a mean MaSD of 4.2–4.9 mm (compared to 2.91–2.95) indicates that the 3D shape could not be reproduced completely by both sizing methods [1]. According to meniscal width, length and height, the 3D-CT sizing method was better than the radiographic method, except for the meniscal height of the lateral meniscus. Although some tissue banks have much more different allografts than only 49, even then, they do not possess always the same 3D meniscus size and a more or less approximation of meniscal width and length of the measured dimensions could increase already existing inaccuracy, as shown in a previous study with an imaginary allograft tissue bank of 138 different menisci [1]. Nevertheless, a reliable sizing method would also detect the best possible 3D meniscus allograft, even in a tissue bank with less variety or rare meniscus shapes.

There is reason why we believe that a further improvement is possible. 3D-CT sizing depends crucially on the cutting planes of the proximal tibia. We deliberately chose an automatized method to exclude inter-rater and inter-reader inaccuracy by manual measurements. The basic idea was that similar tibia plateaus would be matched similar good or similar bad to the average tibia plateau template. But, as mentioned above, the better the anatomical separation of the plateau, the better would be the sizing. Although the intercondylar region was excluded in a standardized fashion, an individual adaptation of the tibia plateau size was not performed. Thereby, a large tibia could probably have some remaining intercondylar region and otherwise smaller tibia some missing parts of the plateau. Further, the different orientation of the medial and lateral tibia plateau was matched by the entire plateau orientation. Thereby, the orientation of medial and lateral plateau would be approximated. But so far, an association of three-dimensional meniscal size and corresponding tibia plateau was never examined in detail. An inter-individual anatomical mismatch is therefore not excluded and could be responsible for part of the inaccuracy.

There are some limitations of this study.

1. Anatomical comparison: Measurements have not been compared with the anatomical meniscus dimensions. We chose 3D meniscus surface models as gold standard, which has not yet been proven. But MRI measurement seems to be the most exact imaging method for meniscus sizing, based on the literature [7, 9, 11, 13, 18].
2. Meniscus segmentation: Because of the retrospective design of the study, MRI had a slice thickness of up to 3 mm, which could falsify the 3D surface model. Therefore, we performed bi-planar segmentation on sagittal and coronal images, as described in a previous study with excellent inter-rater and inter-reader reliability for width and length (ICC_inter_ 0.913–0.973, ICC_intra_ 0.955–0.987, 2].
3. Meniscus shape deformation: The 3D shape of the medial and lateral meniscus changes during knee movement and could falsify our study results. All MRI scans were performed in supine position with full extended knee joint. Therefore, this limitation should be very low.
4. Imaginary “tissue bank”: We validated our method by an imaginary tissue bank of 49 different menisci. A larger tissue bank would have automatically a larger variety of the allografts. Therefore, the accuracy could be improved by an increasing number of different allografts. Nevertheless, there are also tissue banks with only 15–30 different menisci (on request) and a reliable sizing method should also be able to select a good fitting allograft out of a small databank.
5. Correlation of meniscus and tibia plateau size: So far, an inter-individual anatomical difference between the correlation of meniscus and tibia plateau size is not excluded and could be responsible for a part of sizing inaccuracy. Additional inaccuracy could be present in patients with beginning osteoarthritis and alterations of the tibia plateau (osteophytes). This has to be declared as a potential limitation of 3D-CT sizing.

Overall, 3D-CT sizing could be an interesting approach to revolutionize the currently used meniscus sizing methods, which could be especially useful for small companies with limited allografts to increase the accuracy of allograft selection. Although 3D-MRI meniscus sizing by the contralateral side seems to be – at the moment – more precise, 3D-CT sizing could be improved by machine learning and/or individual adjustment of the cutting planes. The advantages would be the independence of the meniscus condition, full automatized with already existing technologies, less cost intensive and faster compared to the 3D-MRI sizing method. But, as already mentioned in previous study [2], the tissue banks have to offer this option, which is associated with higher costs.

## Conclusion

Automatized, indirect meniscus sizing by the 3D bone models of the tibia plateau is feasible and more precise than by the radiographic method of Pollard. This method might become the gold-standard after total meniscectomy if 2D-MRI sizing is not possible. However, further technical improvement is needed to select always the best available allograft.

## Abbreviations

2D: Two-dimensional
3D: Three-dimensional
AP: Antero-Posterior
CT: Computer Tomography
H: Height
i.e.: id est.; that is to say
ICP: Iterative Closest Point
kV: Kilovolt
L: Length
LAT: Lateral
LM: Lateral Meniscus
mAs: Milliampere seconds
MaSD: Maximum Surface Distance
MeSD: Mean Surface Distance
MIMICS: Materialise Interactive Medical Control System
mm: Millimetre
MM: Medial Meniscus
MRI: Magnetic Resonance Imaging
OBB: Oriented Bounding Box
RX: Radiography
Std: Standard Deviation
T: Tesla
W: Width

## References

[1] Beeler S, Jud L, von Atzigen M, Sutter R, Furnstahl P, Fucentese SF (2020). Three-dimensional meniscus allograft sizing-a study of 280 healthy menisci. J Orthop Surg Res.

[2] Beeler Silvan, Vlachopoulos Lazaros, Jud Lukas, Sutter Reto, Fürnstahl Philipp, Fucentese Sandro F. (2019). Contralateral MRI scan can be used reliably for three-dimensional meniscus sizing — Retrospective analysis of 160 healthy menisci. The Knee.

[3] Buma P, Ramrattan NN, van Tienen TG, Veth RP (2004). Tissue engineering of the meniscus. Biomaterials.

[4] Cameron JC, Saha S (1997). Meniscal allograft transplantation for unicompartmental arthritis of the knee. Clin Orthop Relat Res.

[5] Carpenter JE, Wojtys EM, Husten LJ, Crabbe JP, Aisen AM (1993). Preoperative sizing of meniscal allografts: James E. Carpenter, Edward M. Wojtys, Laura J. Huston, Jeffrey P. Crabbe, and Alex M. Aisen. Ann Arbor, Michigan, U.S.A. Arthroscopy.

[6] Dienst M, Greis PE, Ellis BJ, Bachus KN, Burks RT (2007). Effect of lateral meniscal allograft sizing on contact mechanics of the lateral tibial plateau: an experimental study in human cadaveric knee joints. Am J Sports Med.

[7] Donahue TL, Hull ML, Howell SM (2006). New algorithm for selecting meniscal allografts that best match the size and shape of the damaged meniscus. J Orthop Res.

[8] Getgood A, Gelber J, Gortz S, De Young A, Bugbee W (2015). Combined osteochondral allograft and meniscal allograft transplantation: a survivorship analysis. Knee Surg Sports Traumatol Arthrosc.

[9] Haen TX, Boisrenoult P, Steltzlen C, Pujol N (2018). Meniscal sizing before allograft: comparison of three imaging techniques. Knee.

[10] Haut Donahue TL, Hull ML, Rashid MM, Jacobs CR (2004). The sensitivity of tibiofemoral contact pressure to the size and shape of the lateral and medial menisci. J Orthop Res.

[11] Haut TL, Hull ML, Howell SM (2000). Use of roentgenography and magnetic resonance imaging to predict meniscal geometry determined with a three-dimensional coordinate digitizing system. J Orthop Res.

[12] Huang A, Hull ML, Howell SM, Haut Donahue T (2002). Identification of cross-sectional parameters of lateral meniscal allografts that predict tibial contact pressure in human cadaveric knees. J Biomech Eng.

[13] Kaleka CC, Netto AS, Silva JC, Toma MK, de Paula Leite Cury R, Severino NR (2016). Which are the Most reliable methods of predicting the meniscal size for transplantation?. Am J Sports Med.

[14] McConkey M, Lyon C, Bennett DL, Schoch B, Britton C, Amendola A (2012). Radiographic sizing for meniscal transplantation using 3-D CT reconstruction. J Knee Surg.

[15] McDermott ID, Sharifi F, Bull AM, Gupte CM, Thomas RW, Amis AA (2004). An anatomical study of meniscal allograft sizing. Knee Surg Sports Traumatol Arthrosc.

[16] Papalia R, Del Buono A, Osti L, Denaro V, Maffulli N (2011). Meniscectomy as a risk factor for knee osteoarthritis: a systematic review. Br Med Bull.

[17] Pollard ME, Kang Q, Berg EE (1995). Radiographic sizing for meniscal transplantation. Arthroscopy.

[18] Prodromos CC, Joyce BT, Keller BL, Murphy BJ, Shi K (2007). Magnetic resonance imaging measurement of the contralateral normal meniscus is a more accurate method of determining meniscal allograft size than radiographic measurement of the recipient tibial plateau. Arthroscopy.

[19] Rath E, Richmond JC, Yassir W, Albright JD, Gundogan F (2001). Meniscal allograft transplantation. Two- to eight-year results. Am J Sports Med.

[20] Rodeo SA (2001). Meniscal allografts--where do we stand?. Am J Sports Med.

[21] Ryu RK, Dunbar VW, Morse GG (2002). Meniscal allograft replacement: a 1-year to 6-year experience. Arthroscopy.

[22] Shaffer B, Kennedy S, Klimkiewicz J, Yao L (2000). Preoperative sizing of meniscal allografts in meniscus transplantation. Am J Sports Med.

[23] Stevenson Ciara, Mahmoud Ahmed, Tudor Francois, Myers Peter (2019). Meniscal allograft transplantation: undersizing grafts can lead to increased rates of clinical and mechanical failure. Knee Surgery, Sports Traumatology, Arthroscopy.

[24] Szojka A, Lalh K, Andrews SHJ, Jomha NM, Osswald M, Adesida AB (2017). Biomimetic 3D printed scaffolds for meniscus tissue engineering. Bioprinting.

[25] van Arkel ER, de Boer HH (2002). Survival analysis of human meniscal transplantations. J Bone Joint Surg Br.

[26] Yoon JR, Kim TS, Lim HC, Lim HT, Yang JH (2011). Is radiographic measurement of bony landmarks reliable for lateral meniscal sizing?. Am J Sports Med.

[27] Zhang ZZ, Wang SJ, Zhang JY, Jiang WB, Huang AB, Qi YS (2017). 3D-printed poly(epsilon-caprolactone) scaffold augmented with Mesenchymal stem cells for Total meniscal substitution: a 12- and 24-week animal study in a rabbit model. Am J Sports Med.

